# Editorial: Cerebral venous thrombosis

**DOI:** 10.3389/fneur.2022.1021547

**Published:** 2022-09-23

**Authors:** Mirjam R. Heldner, Diana Aguiar de Sousa, Thalia S. Field, Jonathan M. Coutinho, Susanna M. Zuurbier

**Affiliations:** ^1^Department of Neurology, University Hospital Bern, Bern, Switzerland; ^2^Department of Neurology, Lisbon Central University Hospital, Lisbon, Portugal; ^3^Department of Neurology, Faculty of Medicine, University of British Columbia, Vancouver, BC, Canada; ^4^Department of Neurology, Amsterdam University Medical Center, Amsterdam, Netherlands

**Keywords:** cerebral venous thrombosis, vaccine induced immune thrombotic thrombocytopenia, Janus Kinase 2 V617F mutation, inflammation, batroxobin

Cerebral venous thrombosis (CVT) is a cerebrovascular disease that most often affects young adults, especially women. CVT has a varied clinical presentation and can be challenging to recognize. The clinical presentation is frequently subacute, but one-third of patients have an acute onset of their symptoms. The most common symptom is a headache, which is present in more than 90% of patients. Focal neurological deficits occur in approximately half of the patients with CVT. The spectrum of parenchymal damage due to CVT includes a wide range of manifestations, from cerebral edema to venous infarction and intracerebral hemorrhage. Additionally, there is limited evidence on emerging risk factors, such as vaccine-induced immune thrombotic thrombocytopenia (VITT), or the benefits of newer therapies such as endovascular treatment, decompressive craniectomy, and Non-Vitamin K antagonist oral anticoagulants (NOACs). This series focuses on risk factors, clinical characteristics, and therapies for CVT.

One of the major recent developments associated with CVT is VITT. Braun et al. present two cases of ChAdOx1 nCov-19 (AstraZeneca)-associated thrombotic thrombocytopenia syndrome and CVT. Both patients received combined treatment with intravenous immunoglobulins and parenteral anticoagulation, with a subsequent increase in platelet counts for both individuals. Wiedmann et al. present five cases of CVT with thrombocytopenia after a ChAdOx1 nCov-19 vaccination. All female patients presented with an intracerebral hemorrhage. A clinical hallmark was the rapid and severe disease progression despite maximum treatment efforts, resulting in a fatal outcome in four out of five patients. An autopsy was performed in all five cases and showed venous hemorrhagic infarctions and thrombi in dural venous sinuses. The thrombus material was found to be rich in thrombocytes, leukocytes, and fibrin. The vessel walls were free of inflammation. Clinicians should be alert to cases where patients vaccinated with ChAdOx1 nCoV-19 develop symptoms such as increased headaches, neurological deficits, and thrombocytopenia within 4 weeks of receiving this vaccine. An early investigation for VITT is indicated in these cases. For cases of suspected or confirmed VITT, the European Stroke Organization interim expert opinion on CVT occurring after SARS-CoV-2 vaccination is recommended: non-heparin anticoagulants, avoiding platelet transfusions, and early initiation of intravenous immunoglobulin.

In up to 30% of CVT cases, the cause of CVT remains idiopathic. Although CVT is associated with Janus Kinase 2 V617F mutation (JAK-2), the prevalence in CVT cases is unclear. In a retrospective study by Orion et al., the JAK-2 positivity rate was 18% (among 77 patients without additional causes for CVT identified, who were tested for the mutation). Interestingly, they found that patients with normal blood counts on presentation comprised 36% of the JAK-2-positive cases. JAK-2 mutations may be associated with a more complicated clinical course. Thus, they conclude that JAK-2 mutations are underdiagnosed and screening may be warranted in cases of idiopathic CVT, even in patients with normal blood counts. The identification of a JAK-2 mutation diagnosis could change the management of idiopathic CVT, with the potential use of disease-modifying treatment, and ongoing blood cell count monitoring.

The underlying pathogenesis of CVT remains largely unclear. Hu et al. describe increasing evidence suggesting that inflammation contributes to the pathophysiology of severe CVT. Preclinical studies have identified components of neuroinflammation. This includes evidence of activated microglia, which secrete cytokines resulting in blood-brain barrier disruption, brain edema, and cerebral venous infarction. Astrocytes are also activated and may interact with microglia to potentiate the inflammatory response. Some clinical researchers have suggested that inflammation-related biomarkers may be of value in assessing the course, severity, and prognosis of CVT. Future studies including novel anti-inflammatory interventions for severe CVT may potentially be warranted and could contribute to clarifying the pathophysiological mechanisms of severe disease.

In recent years, the role of batroxobin has received attention as a potential novel treatment for CVT. Batroxobin is a medicine with a hemostatic effect. The mechanism of action includes the decomposition of fibrinogen to fibrin degradation products and D-dimer, and the mobilization of endothelial cells to release endogenous rt-PA, promoting thrombolysis. Lan et al. performed a systematic review to summarize current studies of batroxobin in CVT. They found two clinical studies, including 92 clinical cases with CVT, that evaluated the efficacy of combined batroxobin and anticoagulation in CVT. They found higher recanalization rates with batroxobin. In one of the two studies, the National Institutes of Health Stroke Scale scores significantly improved at discharge compared with baseline. An additional study of 13 patients evaluated batroxobin in patients with cortical vein thromboses. Patients on batroxobin had a significantly improved prognosis. Further research examining batroxobin for CVT is warranted; current clinical studies are limited by small sample sizes and high heterogeneity.

In summary, our knowledge of CVT continues to evolve. Our understanding of risk factors is improving over time. Novel therapies targeting inflammation and novel hemostatic pathways may shed light on pathophysiological mechanisms and may potentially contribute to personalized treatments for CVT in the future.

## Author contributions

SMZ wrote the initial draft. All authors contributed to the conceptualization and revision of the manuscript.

## Conflict of interest

The authors declare that the research was conducted in the absence of any commercial or financial relationships that could be construed as a potential conflict of interest.

## Publisher's note

All claims expressed in this article are solely those of the authors and do not necessarily represent those of their affiliated organizations, or those of the publisher, the editors and the reviewers. Any product that may be evaluated in this article, or claim that may be made by its manufacturer, is not guaranteed or endorsed by the publisher.

